# Antibacterial constituents of three Cameroonian medicinal plants: *Garcinia nobilis, Oricia suaveolens* and *Balsamocitrus camerunensis*

**DOI:** 10.1186/1472-6882-13-81

**Published:** 2013-04-10

**Authors:** Hugues Fouotsa, Armelle T Mbaveng, Celine D Mbazoa, Augustin E Nkengfack, Shaheen Farzana, Choudhary M Iqbal, Jacobus J Marion Meyer, Namrita Lall, Victor Kuete

**Affiliations:** 1 Department of Organic Chemistry, Faculty of Science, University of Yaoundé 1, Po.box 812, Yaoundé, Cameroon; 2 Department of Biochemistry, Faculty of Science, University of Dschang, Dschang, Cameroon; 3 Department of Organic Chemistry, Higher Teachers Training College, University of Maroua, P.O.Box 46, Maroua, Cameroon; 4 H. E. J. Research Institute of Chemistry, International Center for Chemical and Biological Sciences, University of Karachi, Karachi 75270, Pakistan; 5 Department of Plant Science, Faculty of Agricultural and Biological Science, Pretoria 0002, South Africa

**Keywords:** Antimycobacterial, Antibacterial, Cameroon, Compounds, *Garcinia nobilis*, *Oricia suaveolens*, *Balsamocitrus camerunensis*

## Abstract

**Background:**

Multidrug resistance is a worrying cause of treatment failure in bacterial infections. The search of bioactive constituents from medicinal plants against multidrug resistant (MDR) bacteria has significantly evolved in the two last decades. In the present study, twenty-two compounds (three terpenoids, eleven phenolics and eight alkaloids) isolated from three Cameroonian medicinal plants, namely *Garcinia nobilis, Oricia suaveolens* and *Balsamocitrus camerunensis*, as well as the crude extracts were tested for their antibacterial activities against *Mycobacterium tuberculosis* and Gram-negative bacteria amongst which were MDR active efflux pumps expressing phenotypes.

**Methods:**

The microplate alamar blue assay (MABA) and the broth microdilution methods were used to determine the minimal inhibitory concentrations (MIC) and minimal bactericidal concentrations (MBC) of the studied samples.

**Results:**

The results of the MIC determinations indicate that, the best crude extract was that from *G. nobilis* (GNB), its inhibitory effects being noted against 12 of the 14 tested bacteria. The extract of GNB also exhibited better anti-tuberculosis (MIC of 128 μg/ml *M. tuberculosis* against ATCC 27294 strain) and antibacterial (MIC of 64 μg/ml against *Escherichia coli* ATCC10536) activities compared to the extracts of *O. suaveolens* and *B. camerunensis*. Interestingly, 4-prenyl-2-(3,7-dimethyl-2,6-octadienyl)-1,3,5,8-tetrahydroxyxanthone (**2**), isolated from the most active extract GNB, also showed the best activity amongst compounds, inhibiting the growth of all the fourteen tested microorganisms. The lowest MIC value obtained with compound **2** was 8 μg/ml against *M. tuberculosis* ATCC 27294 and *M. tuberculosis* clinical MTCS2 strains. Other compounds showed selective activities with 11 of the 14 tested bacteria being sensitive to the xanthone, morusignin I (**5**) and the alkaloid, kokusaginine (**13**).

**Conclusions:**

The results of the present investigation provide evidence that the crude extract from *G. nobilis, O. suaveolens* and *B. camerunensis* as well as some of their compounds, and mostly compound **2** (isolated from *G. nobilis,*) could be considered as interesting natural antibacterial products.

## Background

The continuous emergence of multidrug-resistant (MDR) bacteria drastically reduced the efficacy of our antibiotic armory and, consequently, increases the frequency of therapeutic failure. Drug resistance is a consequence of the worldwide use of antibiotics, and the acute challenge for health care is to find measures to efficiently combat resistant organisms [1]. Several natural compounds have successfully been tested for their abilities to prevent the growth of MDR bacteria [2]. In our continuous search of antibacterial drugs from natural source, we targeted three Cameroonian medicinal plants, *Garcinia nobilis* Engl. (*Clusiaceae*), *Oricia suaveolens* Engl. [Commonly known in West Africa as Abe iolo or Kru-bete parihi (Ivory Coast) or Mende jagbole (Sierra Leone)] and *Balsamocitrus camerunensis* Letouzey, R. (*Rutaceae*). Plants of the genus *Garcinia*, widely distributed in the tropical Africa, Asia, New Caledonia, and Polynesia, have yielded many biologically active and structurally intriguing natural products [3]. *Garcinia* species are known to contain a wide variety of oxygenated and prenylated xanthones, as well as polyisoprenylated benzophenones such as the guttiferones [4]. Previous studies of the chemistry of the genus *Oricia*, including *O. suaveolens* revealed the presence of alkaloids and triterpenes [5,6]. *B. camerunensis* is a new species recently found in Batouri (Cameroon) and Boukoko (Central African Republic) [7]. Plants of genus *Balsamocitrus* (including the decoction of the bark of *B. camerunensis*) are used in traditional African medicine to treat malaria, hypertension, infertility, and influenza [8,9]. Previous phytochemical investigations of this genus revealed the presence of coumarins, quinoline alkaloids, free aliphatic acids and steroids, some of these compounds exhibiting potent antibacterial, fungicidal, and algicidal properties [8]. The aqueous decoctions of the bark of *G. nobilis* as well as that of the roots of *O. suaveolens* are used in Cameroon to treat gastro-intestinal infections [Personal communication]. The combination of the three plants (*G. nobilis*, *O. suaveolens* and *B. camerunensis*) is also used locally to treat stomachache and diarrheal infections [Personal communication]. The present study was therefore designed to evaluate the antibacterial activities of the naturally occurring compounds from *G. nobilis*, *O. suaveolens* and *B. camerunensis* with emphasis on MDR Gram-negative bacteria and *Mycobacterium tuberculosis.*

## Methods

### General experimental procedure

^1^H and ^13^C NMR spectra were recorded in chloroform on Digital NMR BRUCKER AVANCE 400 and 500 MHz. EIMS were obtained on Joel the MS route JMS.600H and HREIMS were performed on a thermo finnigan Mat 95 XP. Thin layer chromatography (TLC) and pre-coated TLC were performed on silica gel GF_254_ (Merck). Column chromatography (CC) was performed on silica gel (Merck) type 100(70–230 Mesh ASTM) eluted either with gradient system (Hex-Ethyl Acetate-MeOH; Hex-CH_2_Cl_2_-MeOH and Hex-CH_2_Cl_2_-Acetone). All the solvents used were distilled commercial grade. The isolated compounds were crystallized from the same solvent and their purity was checked by TLC. Pre-coated plates of silica gel GF_254_ (Merck) were used for this purpose; the spots were detected with UV lamp at 254 and 366 nm and by spraying with 50% H_2_SO_4_ or ceric sulfate following by heating.

### Plant material

The stem bark of the *G. nobilis* was collected in Okola-Yaounde (Center Region of Cameroon) in April, 2010 whilst *O. suaveolens* (woods and stem bark), and *B. camerunensis* (stem bark) were collected in Nkobi village (Batouri, East region of Cameroon), in August 2007. The plants were identified by Mr. Victor Nana of the Cameroon National Herbarium (Yaoundé) where voucher specimen (50779/HNC/Cam for *G. nobilis,* 6161/SRF/Cam for *O. suaveolens* and 3785/SRFK for *B. camerunensis*) were deposited.

### Extraction

The air-dried and powdered samples from each plant were macerated in either 7.0 L methanol (MeOH) for the stem bark of *G. nobilis* (2 kg) or in 10 L MeOH/dichloromethane (CH_2_Cl_2_) mixture for the wood (4 kg) and roots (3 kg) of *O. suaveolens*, and the stem bark (3.8 kg) of *B. camerunensis.* The extraction was done at room temperature for two days. The evaporation under reduced pressure yielded the crude extracts from the stem bark of *G. nobilis* (GNB; 100 g), wood (OSW; 173 g) and roots (OSR; 145 g) from *O. suaveolens*, and from the stem bark *B. camerunensis* (BCB; 128 g).

#### Isolation and identification of compounds from garcinia nobilis

**Figure 1:**
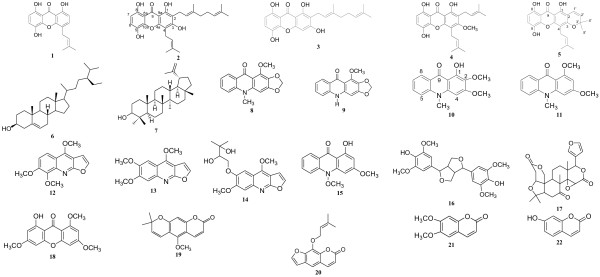
**Chemical structures of compounds isolated from the stem bark of *Garcinia nobilis *(1-5)*, Oricia suaveolens *woods (6-11) and roots (12-17)*, *and the stem back of *Balsmocitrus camerunensis *(18-22).** Caroxanthone (**1**), 4-prenyl-2-(3,7-dimethyl-2,6-octadienyl)-1,3,5,8-tetrahydroxyxanthone (**2**), smeathxanthone A (**3**), 8-hydroxycudraxanthone G (**4**), morusignin I (**5**), stigma-5-en-3-ol (**6**), lupol (**7**), evoxanthine **8**), norevoxanthine (**9**), 1-hydoxy-2,3-dimethoxy-10-methylacridone(**10**), 1,3-dimethoxy-10-methylacridone (**11**), skimmianine (**12**), kokusaginine (**13**), montrifoline (**14**), 1-hydroxy-4-methoxy-10-methylacridone (**15**), syringaresinol (**16**), limonin (**17**), 1-hydroxy-3,6-dimethoxy-8-methylxanthone (**18**), xanthoxyletin (**19**), imperatorin (**20**), 6,8-dimethoxycoumarin (**21**), umbelliferone (**22**).

The compounds from GNB tested herein, caroxanthone (**1**), 3-dimethyl-2-geranyl-4-prenylbellidifolin (**2**), smeathxanthone A (**3**), 8-hydroxycudraxanthone G (**4**) and morusignin I (**5**) (Figure 1) were obtained directly from our chemical bank. We previously reported their isolation and identification from GNB [10].

#### Isolation and identification of compounds from oricia suaveolens

One hundred grams of OSW were submitted to vacuum liquid chromatography (VLC; 25–40 μm, 8 cm × 60 cm, 400 g), eluting with *n*-hexane-Ethyl acetate (EtOAc ) of increasing polarity. Fractions of 500 mL each were collected and subsequently pooled on the basis of their thin layer chromatography (TLC) profile into five main fractions [*n*-hexane- EtOAc 8:2 (2 L, 15 g, fraction 1); 6:4 (2 L, 30 g, fraction 2); 4:6 (1.5 L, 12 g, fraction 3); 2:8 (1.5 L, 15 g, fraction 4); 0:10 (1 L, 10.5 g, fraction 5)]. Fraction 2 (30 g) was then subjected to silica gel 60 column chromatography (25–40 μm, 6 cm × 60 cm, 100 g), eluting with n-hexane-EtOAc mixture of increasing polarity. Sub-fractions (sub-fr) of 100 mL each were collected, then pooled into nine main sub-frs obtained as follows: *n*-hexane- EtOAc 10:0 (1 L, sub-fr A); 9:1 (1.5 L, sub-fr B); 8:2 (1.5 L, sub-fr C); 7.5:2.5 (1 L, sub-fr D); 7:3 (750 mL, sub-fr E); 6:4 (900 mL, sub-fr F); 5:5 (500 mL, sub-fr G); 3:7 (500 mL, sub-fr H) and pure EtOAC (1 L, sub-fr I). Stigma-5-en-3-ol C_29_H_50_O (**6**; 88.3 mg; *m/z:* 414; mp: 250°C) [11] and lupeol C_30_H_50_O (**7**; 57.1 mg; *m/z:* 426; mp: 215-216°C) [12] were obtained from sub-frs A (4.2 g) and B (6.0 g) respectively by recrystallization. Sub-frs A and B residues were then combined based on their similar TLC profile to obtain a new sub-fr named AB (7.5 g). The sub-fr AB was purified on silica gel column (25–40 μm, 4 cm × 60 cm, 80 g) using n-hexane-EtOAc with increasing polarity. Based on their TLC profiles, 15 new sub-frs of 100 mL each were obtained; evoxanthine C_16_H_13_NO_4_ (**8**; 30.0 mg; *m/z:* 283; mp:218-218°C) [13] was obtained by recrystallisation from new sub-frs 3–6 (*n*-hexane- EtOAc 8:2; 7.5:2.5; 7:3; 6.5:3.5) whilst sub-frs 9–12 (*n*-hexane- EtOAc: 5:5; 4.5:5.5; 4:6; 3:7) yielded evoxanthidine C_15_H_11_NO_4_ (**9**; 18.1 mg; *m/z:* 269; mp:310-312°C) by recrystallisation [14].

Sub-frs C-D (7 g) were combined on the basis of their TLC profiles, then subjected to column chromatography over silica gel (25–40 μm 3 cm × 6 cm, 70 g) and eluted with the increasing polarity *n*-hexane- EtOAc to afford ten other sub-frs. Sub-frs 2–4 (obtained with *n*-hexane- EtOAc 7:3; 6:4; 5:5, 2.6 g) yielded 1-hydoxy-2,3-dimethoxy-10-methylacridone or arborinine C_16_H_15_NO_4_ (**10**; 12.1 mg; *m/z:* 285; mp:175-176°C) [15] and subsequent sub-frs 6–8 (obtained *n*-hexane- EtOAc 3:7; 2.5:7.5; 2:8) yielded 1,3-dimethoxy-10-methylacridone C_16_H_13_NO_3_ (**11**; 8.0 mg; *m/z:*269; mp:165°C) [14].

One hundred grams of OSR were partitioned in the water to obtain sequentially hexane fraction (20.1 g), CH_2_Cl_2_ fraction (24.7 g), acetone fraction (17.0 g) and the MeOH fraction (32.5 g). The CH_2_Cl_2_ crude extract (24.7 g) was then submitted to VLC on silica gel (25–40 μm, 8 cm × 60 cm, 100 g) and eluted with hexane-ethyl acetate gradients: (8:2, 1.5 L), (6:4, 1.5 L), (5:5, 1.25 L), (4:6, 1.25 L), (2:8, 1 L) and finally with pure EtOAc (500 mL) to give 40 fractions of 250 ml each. These fractions were pooled on the basis of their TLC profiles. Fractions 1–17 (10 g), obtained with 80% hexane–EtOAc were subjected to column chromatography over silica gel (25–40 μm, 4 cm × 60 cm, 65 g) eluting with hexane-CH_2_Cl_2_ with a continuous gradient (95:5 to 7:3) to yield skimmianine C_14_H_13_NO_4_ (**12**; 2.1 mg; *m/z:* 259; mp:176-177°C) [16] and kokusaginine C_14_H_13_NO_4_ (13; 7.5 mg; *m/z:* 259; mp:171°C) [16]. A continuous elution by increasing the solvent polarity (6:4 to pure CH_2_Cl_2_) yielded four mixtures sub-frs (A-D). Sub-fr B (2.7 g), showed after examination on TLC precoated plate a mixture of two compounds. This fraction was further purified on silica gel column chromatography (25–40 μm; 3 × 15 cm, 15 g) eluting with hexane-CH_2_Cl_2_ with a continuous gradient (6:4 to 3:7) to yield montrifoline C_18_H_21_NO_6_ (**14**; 2.7 mg, *m/z:* 347; mp:1190-192°C) [17] and 1-hydroxy-3-methoxy-10-methylacridone C_15_H_13_NO_3_ (**15**; 15.0 mg; *m/z:* 255; mp:164–165; 174-176°C) [18]. Sub-fr C (2.55 g) was then subjected to silica gel column chromatography eluting with hexane-CH_2_Cl_2_ with a continuous gradient (6:4 to pure CH_2_Cl_2_) to yield (+/−)syringaresinol C_22_H_26_O_8_ (**16**; 9.2 mg; *m/z:* 418; mp:185-186°C; mp:1175-176°C) [19] whilst combined sub-frs 21–40 (25 g) obtained from the (1:1) to (1:3) hexane–EtOAc mixtures and pure EtOAc were subjected successively to silica gel column chromatography and preparative TLC, eluting with solvent mixtures (hexane-CH_2_Cl_2_-EtOAc) of increasing polarity to yield limonin C_26_H_30_O_8_ (**17**; 12.0 mg; *m/z:* 470; mp:298°C) [20].

#### Isolation and identification of compounds from balsamocitrus camerunensis

One hundred and twenty grams of BCB were partitioned by dissolving in CH_2_Cl_2_ to give a soluble (42.0 g) and insoluble (76.5 g) fractions. The CH_2_Cl_2_ soluble fraction was subjected to silica gel VLC (25–40 μm, 8 cm × 60 cm, 120 g) and eluted with the increasing polarity of *n-*hexane-EtOAc and MeOH to afford 160 sub-frs of 250 mL each. These sub-frs were pooled on the basis of TLC analysis into four main fractions (A-D).

Fraction A (12.5 g, combined from sub-frs 1–40) was column chromatographed over silica gel (25–40 μm, 3.5 cm × 50.0 cm) with *n*-hexane-CH_2_Cl_2_ gradient. A total of 40 new sub-fractions of 100 mL each were collected and pooled on the basis of the TLC analysis. Sub-fr 5–15 were further column chromatographed over silica gel (25–40 μm, 2.5 cm × 30.0 cm), using *n*-hexane-CH_2_Cl_2_ (4:1) mixture as eluent to afford compound 7 (99 mg). Sub-frs 16–40 were further chromatographed over silica gel column (25–40 μm, 2.5 cm × 30.0 cm), eluting with *n*-hexane-CH_2_Cl_2_ (1:1) mixture to yield 1-hydroxy-3,6-dimethoxy-8-methylxanthone or lichexanthone C_17_H_16_O_4_ (**18**; 5.2 mg; *m/z:* 284; mp:187-188°C) [21] and xanthoxyletin C_15_H_14_O_4_ (**19**; 7.0 mg; *m/z:* 258; mp:133°C) [22]. Fraction B (10.0 g, combined from the sub-frs 41–79) was purified over silica gel column (25–40 μm, 3.5 × 50.0 cm) with *n*-hexane-CH_2_Cl_2_ gradient. A total of 50 sub-frs of 100 mL each were collected and pooled on the basis of TLC analysis. Sub-frs 10 to 40 were further purified over silica gel column (25–40 μm, 2.5 cm × 30.0 cm) with a mixture of *n*-hexane-CH_2_Cl_2_ (9 : 1) to yield imperatorin C_16_H_14_O_4_ (**20**;12.0 mg, *m/z:* 270; mp:102°C) [23]. Fraction C (16.5 g, combined from sub-frs 80–110) was purified over silica gel column (25-40 μm, 3.5 cm × 50.0 cm) with a Hexane- CH_2_Cl_2_ gradient. A total of 45 fractions of 100 mL each were collected and combined on the basis of their TLC profile. Fractions 10–25 were further purified over silica gel column (25–40 μm, 2.5 cm × 30.0 cm) with CH_2_Cl_2_-acetone (4: 1) mixture to afford scoparone C_11_H_10_O_4_ (**21**; 8.7 mg; *m/z:* 206; mp: 144°C) [24]. Fraction D was further chromatographed on silica gel column, using CH_2_Cl_2_–acetone mixture as eluent, then the recrystallization in CH_2_Cl_2_-acetone (6:4) gave umbelliferone C_9_H_6_O_3_ (**22**; 11.0 mg; *m/z:* 162; mp:230-232°C) [25].

### Chemicals for antibacterial assay

Chloramphenicol (Sigma-Aldrich, St. Quentin Fallavier, France) was used as reference antibiotics (RA) against Gram-negative bacteria. *p*-Iodonitrotetrazolium chloride (INT, Sigma-Aldrich) was used as microbial growth indicator [26,27]. Isoniazid (INH) (Sigma) was used as RA against *M. tuberculosis*.

### Antibacterial assays

#### Microbial strains and culture media

The studied microorganisms included reference and clinical strains (Tables 1 and 2) of *Escherichia coli, Enterobacter aerogenes, Klebsiella pneumoniae, Providencia stuartii, Pseudomonas aeruginosa,* a drug-susceptible strain of *M. tuberculosis* H37Rv obtained from the American Type Culture Collection, and two clinical strains of *M. tuberculosis* MTCS1, MTCS2. *M. tuberculosis* was plated on Löwenstein–Jensen medium and allowed to grow for 3–4 weeks at 37°C. Middlebrook 7H9 broth supplemented with 0.2% glycerol and 10% Oleic Acid–Albumin–Dextrose–Catalase (OADC) (Sigma) was used to determine the MIC and MBC values of the test samples on *M. tuberculosis*. We previously reported the features [28] of all the tested Gram-negative bacteria. Nutrient agar was used for the activation of bacteria other than *M. tuberculosis* strains [28]. The clinical strains used in this work are our laboratory collection previously obtained from Yaoundé General Hospital (Cameroon), and from the Mediterranean University (Marseille, France).

**Table 1:** **MICs of the extracts, compounds from *Garcinia nobilis, Oricia suaveolens*, *Balsmocitrus camerunensis *and reference drugs on documented strains and clinical MDR isolates**

**Tested samples**^**a**^	**Microorganisms, strains and MIC (μg/ml)**^**b**^														
	***E. coli***			***E. aerogenes***			***K. pneumoniae***		***P. stuartii***		***P. aeruginosa***		***M. tuberculosis***		
	***ATCC 10536***	***AG 100***	***AG 102***	***ATCC 13048***	***CM 64***	***EA 27***	***ATCC 11296***	***KP55***	***ATCC 29916***	***NAE 16***	***PA 01***	***PA 124***	***ATCC 27294***	***MTCS1***	***MTCS2***
Crude extracts															
GNB	64	256	256	256	512	256	128	512	512	-	512	-	128	256	256
OSR	128	512	-	512	-	-	256	256	512	256	-	-	512	-	512
OSW	128	512	-	-	-	-	128	128	-	128	-	-	256	-	512
BCB	512	-	-	256	-	-	512	512	-	-	-	-	-	-	-
Compounds															
1	256	128	-	-	-	-	-	-	-	-	-	-	-	-	-
2	64	64	128	128	256	64	512	512	256	64	64	128	8	32	8
3	128	128	64	256	-	256	-	-	-	-	-	-	64	-	128
4	-	64	128	-	256	128	-	-	-	-	256	-	512	-	512
5	64	128	128	-	-	512	512	-	512	-	64	128	32	64	64
8	512	128	256	-	512	128	512	-	-	-	-	-	128	512	256
9	-	128	256	-	256	256	-	-	-	-	-	-	-	-	-
10	64	128	256	-	-	128	-	-	512	-	-	-	128	-	512
11	128	256	128	-	256	512	512	-	-	-	-	512	-	-	-
13	512	64	256	-	512	128	256	-	-	-	128	512	256	512	512
15	128	256	256	-	-	256	-	-	-	-	-	-	-	-	-
16	128	128	-	-		512	-	-	512	-	-	-	512	-	-
17	-	128	-	-	-	-	-	-	-	-	-	-	-	-	-
18	-	256	-	-	-	256	-	-	-	256	-	-	-	-	-
19	-	256	-	-	-	-	-	-	-	-	-	-	-	-	-
20	-	512	-	-	-	-	-	-	-	-	-	-	-	-	-
21	512	256	-	-	-	256	256	-	-	-	-	-	-	-	-
22	512	256	256	512	128	-	-	512	-	-	-	-	-	-	-
RA	<4	16	512	64	-	64	4	32	-	64	128	128	1	128	4

^a^Tested samples [methanol-dichloromethane 1:1 extract of the stem bark of *Garcinia nobilis* (GNB)*,* the wood (OSW)roots (OSR) of *Oricia suaveolens*, and stem bark of *Balsmocitrus camerunensis* (BCB); compounds isolated from the stem bark of *Garcinia nobilis* (**1**–**5**)*, Oricia suaveolens* woods (**6**–**11**) and roots (**12**–**17**)*,* and the stem back of *Balsmocitrus camerunensis* (**18**–**22**); caroxanthone (**1**), 4-prenyl-2-(3,7-dimethyl-2,6-octadienyl)-1,3,5,8-tetrahydroxyxanthone (**2**), smeathxanthone A (**3**), 8-hydroxycudraxanthone G (**4**), morusignin I (**5**), stigma-5-en-3-ol (**6**), 20(29)lupen-2-ol (**7**), evoxanthine (**8**), norevoxanthine (**9**), 1-hydoxy-2,3-dimethoxy-10-methylacridone (**10**), 1,3-dimethoxy-10-methylacridone (**11**), kokusaginine (**13**), 1-hydroxy-4-methoxy-10-methylacridone (**15**), syringaresinol (**16**), limonin (**17**), 1-hydroxy-3,6-dimethoxy-8-methylxanthone (**18**), xanthoxyletin (**19**), imperatorin (**20**), 6,8-dimethoxycoumarin (**21**), umbelliferone (**22**)]; RA: reference antibiotics were chloramphenicol for bacteria, isoniazid for *M. tuberculosis*; ^b^(−): MIC > 512 μg/mL.

**Table 2:** **MBCs of the extracts, compounds from *Garcinia nobilis, Oricia suaveolens*, *Balsmocitrus camerunensis *and reference drugs on documented strains and clinical MDR isolates**

**Tested samples**^**a**^	**Microorganisms, strains and MBC (μg/ml)**^**b**^														
	***E. coli***			***E. aerogenes***			***K. pneumoniae***		***P. stuartii***		***P. aeruginosa***		***M. tuberculosis***		
	***ATCC 10536***	***AG 100***	***AG 102***	***ATCC 13048***	***CM 64***	***EA 27***	***ATCC 11296***	***KP55***	***ATCC 29916***	***NAE 16***	***PA 01***	***PA 124***	***ATCC 27294***	***MTCS1***	***MTCS2***
**Crude extracts**															
GNB	256	512	>512	>512	>512	>512	256	>512	>512	-	>512	-	256	>512	512
OSR	256	>512	-	>512	-	-	512	512	>512	512	-	-	>512	-	>512
OSW	512	>512	-	-	-	-	256	512	-	512	-	-	512	-	>512
BCB	>512	-	-	512	-	-	>512	>512	-	-	-	-	-	-	-
**Compounds**															
**1**	>512	>512	-	-	-	-	-	-	-	-	-	-	-	-	-
**2**	128	256	256	256	>512	128	>512	>512	512	128	128	256	16	64	32
**3**	256	256	128	512	-	512	-	-	-	-	-	-	128	-	256
**4**	-	128	256	-	512	256	-	-	-	-	512	-	>512	-	>512
**5**	128	256	256	-	-	>512	>512	-	>512	-	128	256	64	128	128
**8**	>512	256	>512	-	>512	256	>512	-	-	-	-	-	256	>512	512
**9**	-	256	>512	-	512	>512	256	-	-	-	-	-	-	-	-
**10**	256	256	512	-	-	-	-	-	>512	-	-	-	512	-	>512
**11**	>512	-	-	-	-	>512	-	-	-	-	-	-	-	-	-
**13**	>512	128	512	-	>512	256	512	-	-	-	256	>512	512	>512	>512
**15**	256	512	>512	-	-	512	-	-	-	-	-	-	-	-	-
**16**	256	256	-	-		>512	-	-	>512	-	-	-	>512	-	-
**17**	-	256	-	-	-	-	-	-	-	-	-	-	-	-	-
**18**	-	512	-	-	-	512	-	-	-	512	-	-	-	-	-
**19**	-	512	-	-	-	-	-	-	-	-	-	-	-	-	-
**20**	-	>512	-	-	-	-	-	-	-	-	-	-	-	-	-
**21**	>512	512	-	-	-	512	512	-	-	-	-	-	-	-	-
**22**	>512	512	512	>512	256	-	-	>512	-	-	-	-	-	-	-
**RA**	<4	32	>512	128	-	128	8	64	-	128	256	256	2	256	16

^a^Tested samples [methanol-dichloromethane 1:1 extract of the stem bark of *Garcinia nobilis* (GNB)*,* the wood (OSW)roots (OSR) of *Oricia suaveolens*, and stem bark of *Balsmocitrus camerunensis* (BCB); compounds isolated from the stem bark of *Garcinia nobilis* (**1**–**5**)*, Oricia suaveolens* woods (**6**–**11**) and roots (**12**–**17**)*,* and the stem back of *Balsmocitrus camerunensis* (**18**–**22**); caroxanthone (**1**), 4-prenyl-2-(3,7-dimethyl-2,6-octadienyl)-1,3,5,8-tetrahydroxyxanthone (**2**), smeathxanthone A (**3**), 8-hydroxycudraxanthone G (**4**), morusignin I (**5**), stigma-5-en-3-ol (**6**), 20(29)lupen-2-ol (**7**), evoxanthine (**8**), norevoxanthine (**9**), 1-hydoxy-2,3-dimethoxy-10-methylacridone (**10**), 1,3-dimethoxy-10-methylacridone (**11**), kokusaginine (**13**), 1-hydroxy-4-methoxy-10-methylacridone (**15**), syringaresinol (**16**), limonin (**17**), 1-hydroxy-3,6-dimethoxy-8-methylxanthone (**18**), xanthoxyletin (**19**), imperatorin (**20**), 6,8-dimethoxycoumarin (**21**), umbelliferone (**22**)]; RA: reference antibiotics were chloramphenicol for bacteria, isoniazid for *M. tuberculosis*; ^b^(−): MBC not determined as the MIC was above 512 μg/ml.

#### INT colorimetric assay for MIC and MBC determinations

The MIC determinations on Gram-negative bacteria were conducted using rapid INT colorimetric assay according to previously described methods [26,27] with some modifications. The test samples and RA were first of all dissolved in DMSO/Mueller Hinton Broth (MHB) or DMSO/7H9 broth. The final concentration of DMSO was lower than 2.5% and does not affect the microbial growth [29]. The solution obtained was then added to MHB, and serially diluted two fold (in a 96- wells microplate). Then, 100 μl of inoculum 1.5 × 10^6^ CFU/ml prepared in appropriate broth was added [30]. The plates were covered with a sterile plate sealer, then agitated to mix the contents of the wells using a plate shaker and incubated at 37°C for 18 h. The assay was repeated thrice. Wells containing adequate broth, 100 μl of inoculum and DMSO to a final concentration of 2.5% served as negative control. The MIC of samples was detected after 18 h incubation at 37°C, following addition (40 μl) of 0.2 mg/ml INT and incubation at 37°C for 30 minutes. Viable bacteria reduced the yellow dye to a pink. MIC was defined as the sample concentration that prevented this change and exhibited complete inhibition of microbial growth. The MBC was determined by adding 50 μl aliquots of the preparations, which did not show any growth after incubation during MIC assays, to 150 μl of adequate broth. These preparations were incubated at 37°C for 48 h. The MBC was regarded as the lowest concentration of extract, which did not produce a color change after addition of INT as mentioned above [30,31].

#### Microplate Alamar blue assay (MABA) against M. Tuberculosis

The activity of all samples against *M. tuberculosis* strains was tested using the MABA [32]. Briefly, each of the above *M. tuberculosis* strains was cultured at 37°C in Middlebrook 7H9 broth supplemented with 0.2% glycerol and 10% OADC (oleic acid–albumin–dextrose–catalase; Sigma) until logarithmic growth was reached. The homogenous culture was obtained using sterile glass beads and vortex. About 6×10^6^ CFU/ml inoculum (100 μl) of *M. tuberculosis* was then added to the two fold serially diluted samples (100 μl). The final concentration of DMSO in all assays was 2.5% or less and this dilution also served as solvent control. The samples were assayed in triplicate. All tests were carried out in sterile flat-bottomed 96-well microplates. Each microplate was incubated for 5 days at 37°C in a sealed plastic CO_2_-permeable bag. After 5 days of incubation, 32 μl of a mixture of freshly prepared Alamar Blue solution and 20% sterile Tween-80 (Sigma) 1:1 v/v were added to one growth-control well. The microplates were incubated again at 37°C for 24 h. If a color shift from blue to pink was observed in the growth-control sample, 32 μl of alamar blue solution was added to each of the remaining wells, and the microplate was further incubated for 24 h. A well-defined pink color was interpreted as positive bacterial growth, whereas a blue color indicated an absence of growth. The MIC corresponded to the greatest dilution of sample extract in which the color shift from blue to pink was not observed.

Samples with recorded MIC values following MABA were assayed for their mycobactericidal effect [32]. Briefly, 5 μl of the undeveloped mycobacterial suspensions were transferred from the former to a new microplate containing 195 μl of fresh culture medium per well. Three wells were inoculated with 100 μl of fresh inoculum as for MABA and three more wells were incubated with 200 μl of culture medium only, as negative controls. The microplates were incubated and developed with alamar blue as for MABA. The MBC corresponded to the minimum sample concentration that did not cause a color shift in cultures that were re-incubated in fresh medium.

## Results and discussion

The chemical structures of the compounds isolated from *G. nobilis, O. suaveolens* and *B. camerunensis* (Figure 1) were established by spectroscopic methods. The compounds were isolated from the stem bark of *G. nobilis* (**1–5**)*,* the woods of *O. suaveolens* (**6–11**), the roots (**12–17**) and the stem bark of *B. camerunensis* (**7**, **18–22**). The twenty two isolated compounds were identified as caroxanthone (**1**), 4-prenyl-2-(3,7-dimethyl-2,6-octadienyl)-1,3,5,8-tetrahydroxyxanthone (**2**), smeathxanthone A (3), 8-hydroxycudraxanthone G (**4**), morusignin I (**5**), stigma-5-en-3-ol (**6**), lupeol (**7**), evoxanthine (**8**), norevoxanthine (**9**), 1-hydoxy-2,3-dimethoxy-10-methylacridone(10), 1,3-dimethoxy-10-methylacridone (11), skimmianine (**12**), kokusaginine (**13**), montrifoline (**14**), 1-hydroxy-4-methoxy-10-methylacridone (**15**), syringaresinol (**16**), limonin (**17**), 1-hydroxy-3,6-dimethoxy-8-methylxanthone (**18**), xanthoxyletin (**19**), imperatorin (**20**), scoparone (**21**) and umbelliferone (**22**) [11-25]. Amongst the twenty-two compounds were three terpenoids (**6, 7** and **17**), eleven phenolic compounds (**1–5, 16, 18–22**), and eight alkaloids (**10–17**). The isolated terpenoids were steroid (**6**), triterpenoid (**7**), and limonoid (**17**) whilst the alkaloids included five acridones (**8–11, 15**) and three furanoquinolines (**12–14**). The phenolics obtained herein were six xanthones (**1–5, 18**), one lignan (**16**) and four coumarins (**19–22**). The isolation and identification of compounds **1–5** from *G. nobilis* was previously reported [10]. The occurrence of alkaloids and terpernoids from *O. suaveolens* was reported [5,6], and their isolation in the present study is in consistence with previous reports. The occurrence of coumarins, quinoline alkaloids, and free aliphatic acids was also reported in *B. camerunensis*[9]. However, in this study, only coumarins were isolated. The crude extracts as well as the isolated compounds [excluding compounds **6, 7, 12** (known to have low or no antimicrobial activity), and **14** (isolated in very low quantities)] were tested for the antibacterial activities against Gram-negative bacteria and *M. tuberculosis* and the results are summarized in Tables 1 and 2.

The results of the MIC determinations (Table 1) showed that the crude extract from *G. nobilis* (GNB) was the most active amongst the studied extracts, its inhibitory effects being noted on 12 of the 14 tested bacteria. GNB also exhibited the best activity against *M. tuberculosis* ATCC 27294 (MIC of 128 μg/ml) and *E. coli* ATCC10536 (MIC of 64 μg/ml) than OSR, OSW and BCB. The inhibitory effects of the extracts OSR and OSW from *O. suaveolens* were noted on 9/14 and 7/14 studied bacteria respectively, meanwhile that of the extract BCB of *B. camerunensis* was observed on 4/14 pathogens tested. Interestingly, compound **2** isolated from the most active extract GNB, also exhibited the best activity, preventing the growth of all the fourteen tested microorganisms. The lowest MIC obtained with compound **2** was 8 μg/ml against *M. tuberculosis* ATCC 27294 and the clinical MTCS2 strains. It is noteworthy that compound **2** was more active than chloramphenicol on two Gram-negative MDR bacteria, namely *E. coli* AG102 and *E. aerogenes* CM64 (Table 1). Other compounds showed selective activities, their effects being noted on 1/14 tested bacteria for compounds **16**, **17**, **19** and **20**; 2/14 for 1; 3/14 for 18; 4/14 for **9** and **21**; 5/14 for **22**; 7/14 for **3**, **4**, **10** and 11; 9/14 for 8; and 11/14 for **5** and **13**. The results of the MBC determinations (Table 2) also showed the activities of the studied samples on some of the tested microorganisms. As observed with MIC data (Table 1), the lowest MBC value (Table 2) was recorded with compound **2** (16 μg/ml) against *M. tuberculosis* ATCC 27294.

Phytochemicals are routinely classified as antimicrobials on the basis of susceptibility tests that produce MIC in the range of 100 to 1000 μg/ml [33]. The activity is considered to be significant if MIC values are below 100 μg/ml for crude extract and moderate when the MICs vary from 100 to 625 μg/ml [34,35]. Also, the activity of compounds is considered to be significant when the MIC is below 10 μg/ml, and moderate when such values vary between 10 and 100 μg/ml [34,35]. On the basis of such criteria, the activity of the studied crude extracts can mostly be considered as moderate, though a significant effect was observed with GNB on *E. coli* ATCC strains. Compound **2** was significantly active against *M. tuberculosis* ATCC 27294 and MTCS2 strains. However its activities can also be considered as moderate against the majority of the bacteria tested. All the tested compounds were active on at least one of the studied microorganisms, and their presence can explain the activity of the crude extracts. Nonetheless, it can be observed that the activity of GNB was not detected on both *P. stuartii* ATCC NAE16 and *P. aeruginosa* PA124, while the extract yielded at least one active compound on these bacteria (Table 1). This can be explained by the fact that the activity of the crude extract does not only depend on the presence of the active compounds, but is also influenced by the quantity and/or possible interaction with other constituents of the plant. This observation can also be applied when carefully analysing the activities of the crude extracts from *O. suaveolens* and *B. camerunensis* and their constituents (Table 1). The activity of the crude extracts and compounds studied herein (and mostly compound **2**) can be considered interesting when regarding the medical importance of the studied bacteria. In fact, the clinical MDR Gram-negative bacteria tested express active efflux pumps and are involved in many therapeutic failures [28]. To the best of our knowledge, the antibacterial activities of the extracts of *G. nobilis, O. suaveolens* and *B. camerunensis* as well as those of most of the studied compounds are being reported for the first time. Nevertheless, some of the isolated compounds were reported for their antibacterial properties. In effect, lupeol (**7**) is known to have low antibacterial activities, the lowest MIC value obtained against *Enterococcus faecalis* ATCC 29212 being 63 μg/ml [36]. The activity of compound **7** was also not detected against the sensitive *Mycobacterium smegmatis* when it was tested at a concentration up to 312 μg/ml [37] and consequently, this compound was not tested again in this work. Previously we also reported the moderate activity of the alkaloid **13** on some sensitive Gram-negative bacteria as well as it low effect against *M. smegmatis*[37]. This compound was not more active against MDR bacteria as observed in the present work, confirming its low antibacterial potential. It has also been demonstrated that compounds **3** (active only on two of 21 tested microorganisms) [38] and **16** (inhibition zone diameters for 10 μl discs at 10^4^ ppm reported as 0.0; 0.2; 0.4 and 0.5 mm against *K. pneumoniae, S. aureus, Pseudomonas syringae* and *Bacillus subtilis* respectively) [39] have low antibacterial activities. Such reports are in accordance with the results obtained in the present study.

Though compounds **17** (limonin) and **20** (imperatonin) showed poor activities as reported herein, their antilisterial inhibitory effects were found moderate, the MIC values obtained against five *Listeria monocytogenes* ATCC strains varying between 15.62-31.25 μg/ml [40].

Compounds **19** (xanthoxyletin) and **22** (umbelliferone) were also found inactive against *M. tuberculosis* and poorly active against Gram-negative bacteria, consolidating the previously reported data [41,42]. It is noteworthy that compound **12** was reported not to have any bacterial activity [43] and was not tested in the present work.

When regarding the structure-activity relationship, it appears that coumarins had the lowest activities, none of them been active against *M. tuberculosis*. The tested tetranortriterpenoid, compound **17** also showed very weak antibacterial activities. These results are in consistence with previous studies, showing the low antibacterial activities of terpenoids and coumarins against bacteria expressing MDR phenotype [2]. Alkaloids also showed low antibacterial activities. However moderate inhibitory effects were noted with **10** (1-hydoxy-2,3-dimethoxy-10-methylacridone) and **13** (kokusaginine) respectively against *E. coli* ATTC and AG100 strains (Table 1). Amongst alkaloids, compound **13**, one of the three isolated furanoquinolines, showed the best spectrum of activity (contrary to the five acridone alkaloids) and was found active on both *M. tuberculosis* and Gram-negative bacteria. Within the acridone alkaloids, and when comparing the effects of compounds **10** and **15** [antibacterial spectra (7/14 of the tested bacteria for 10 and 4/14 for **15**); lowest MIC value (64 μg/ml for 10 and 128 μg/ml for **15**)], it appears that the presence of methoxyl- (−OCH_3_) group in C2 (compound **10**) increases the bacterial susceptibility (Figure 1, Table 1). A comparison of the activities of compounds **8** and **9** [the presence of –OCH_3_ group in C1 (compound **8**) instead of –OH group (compound **9**)] on one hand, and those of **11** and **15** [the presence of –OCH_3_ group in C1 (compound **11**) instead of the hydroxyl (−OH) group (compound **15**)] on another hand seems to confirm the fact that the natural substitution of –OCH_3_ by –OH in the studied alkaloids increases their activities (Figure 1, Table 1). The best antibacterial activities were recorded with xanthones. Within the studied xanthones and when regarding the activities of compounds **2** and **3**, it appears that the presence of additional prenyl- group in C4 significantly increases the antibacterial activity (Figure 1, Table 1). Between compounds **4** and **5**, it can also be deduced that the cyclisation increase the activity (Figure 1, Table 1).

## Conclusion

The results of the present investigation are important, in regards to the medical importance of the studied microorganisms. Hence, these data provide evidence that some of the constituents of *G. nobilis, O. suaveolens* and *B. camerunensis* and mostly compound **2** (isolated from *G. nobilis*), as well as some of the crude extracts from the three plants could be potential antimicrobial products to fight MDR bacteria. The combination of the three plants as used locally in the treatment of infections will further be investigated to provide better understanding to the traditional use.

## Competing interests

The authors declare that they have no competing interests.

## Authors’ contributions

HF, ATM and VK carried out the study; HF and VK wrote the manuscript; VK, AEN, CDM, SF, MIC, JJMM and NL supervised the work. All authors read and approved the final manuscript.

## Pre-publication history

The pre-publication history for this paper can be accessed here:

http://www.biomedcentral.com/1472-6882/13/81/prepub
